# Traffic-Related Air Pollution and Dementia Incidence in Northern Sweden: A Longitudinal Study

**DOI:** 10.1289/ehp.1408322

**Published:** 2015-07-31

**Authors:** Anna Oudin, Bertil Forsberg, Annelie Nordin Adolfsson, Nina Lind, Lars Modig, Maria Nordin, Steven Nordin, Rolf Adolfsson, Lars-Göran Nilsson

**Affiliations:** 1 Occupational and Environmental Medicine, Department of Public Health and Clinical Medicine, Umeå University, Umeå, Sweden; 2 Division of Psychiatry, Department of Clinical Sciences, and; 3 Department of Psychology, Umeå University, Umeå, Sweden; 4 ARC (Aging Research Centre), Karolinska Institutet, Stockholm, Sweden; 5 Umeå Center for Functional Brain Imaging, Umeå University, Umeå, Sweden

## Abstract

**Background:**

Exposure to ambient air pollution is suspected to cause cognitive effects, but a prospective cohort is needed to study exposure to air pollution at the home address and the incidence of dementia.

**Objectives:**

We aimed to assess the association between long-term exposure to traffic-related air pollution and dementia incidence in a major city in northern Sweden.

**Methods:**

Data on dementia incidence over a 15-year period were obtained from the longitudinal Betula study. Traffic air pollution exposure was assessed using a land-use regression model with a spatial resolution of 50 m × 50 m. Annual mean nitrogen oxide levels at the residential address of the participants at baseline (the start of follow-up) were used as markers for long-term exposure to air pollution.

**Results:**

Out of 1,806 participants at baseline, 191 were diagnosed with Alzheimer’s disease during follow-up, and 111 were diagnosed with vascular dementia. Participants in the group with the highest exposure were more likely than those in the group with the lowest exposure to be diagnosed with dementia (Alzheimer’s disease or vascular dementia), with a hazard ratio (HR) of 1.43 (95% CI: 0.998, 2.05 for the highest vs. the lowest quartile). The estimates were similar for Alzheimer’s disease (HR 1.38) and vascular dementia (HR 1.47). The HR for dementia associated with the third quartile versus the lowest quartile was 1.48 (95% CI: 1.03, 2.11). A subanalysis that excluded a younger sample that had been retested after only 5 years of follow-up suggested stronger associations with exposure than were present in the full cohort (HR = 1.71; 95% CI: 1.08, 2.73 for the highest vs. the lowest quartile).

**Conclusions:**

If the associations we observed are causal, then air pollution from traffic might be an important risk factor for vascular dementia and Alzheimer’s disease.

**Citation:**

Oudin A, Forsberg B, Nordin Adolfsson A, Lind N, Modig L, Nordin M, Nordin S, Adolfsson R, Nilsson LG. 2016. Traffic-related air pollution and dementia incidence in northern Sweden: a longitudinal study. Environ Health Perspect 124:306–312; http://dx.doi.org/10.1289/ehp.1408322

## Introduction

The cardiopulmonary effects of air pollutants are well established (Rückerl et al. 2011), and there is a growing body of evidence indicating that air pollution causes neuropathological effects and central nervous system disease (Block and Calderón-Garcidueñas 2009; Guxens and Sunyer 2012; Rückerl et al. 2011; Genc et al. 2012). Dementia is a neuropathological disease that is relevant in this context (Weuve 2014). Dementia takes a heavy toll on the patient, the patient’s close relatives, and society as a whole. Within the next 40 years, the prevalence of Alzheimer’s disease (AD) is expected to triple unless preventive measures are developed (Hebert et al. 2013).

The number of studies suggesting an association between traffic pollution and cognitive function in adults is increasing. In a cross-sectional study of 399 elderly women in Germany, the exposure to traffic-related particles was estimated by the distance to the closest busy road, and consistent associations between traffic-related particle exposure and mild cognitive impairment were found (Ranft et al. 2009). In another cross-sectional study of 1,764 adults in the United States, ozone levels in the participants’ home counties were associated with inferior performance on neurobehavioral tests (Chen and Schwartz 2009). In a third cross-sectional study (of 680 elderly men in the United States), long-term exposure to traffic-related air pollution was associated with lower Mini Mental State Examination (MMSE) scores as well as with lower global cognitive function (Power et al. 2011). A similar study of 765 community-dwelling senior citizens showed residential proximity to a major roadway to be associated with poorer performance on cognitive tests, but weaker associations with modeled outdoor levels of black carbon were observed (Wellenius et al. 2012). In the Nurses’ Health Study Cognitive Cohort, which included 19,409 elderly women in the United States, long-term exposure to particles preceding baseline cognitive testing was assessed (Weuve et al. 2012a). The main outcome measure was cognition, which was assessed via validated telephone interviews at approximately 2-year intervals. Long-term exposure was found to be associated with faster cognitive decline, and a 10-μg/m^3^ increment in long-term particulate matter (both PM_2.5_ and PM_2.5–10_) exposure was cognitively equivalent to aging by approximately 2 years. A cross-sectional study conducted in the Los Angeles Basin in southern California examined the associations between modeled air pollution levels at home and cognitive function in middle-aged and older persons, but no significant associations were found between NO_2_ levels and cognitive function (Gatto et al 2014). The results from a study conducted in Taiwan using data from a national health insurance database of persons ≥ 50 years of age suggested that high concentrations of nitrogen dioxide (NO_2_) and carbon monoxide (CO) concentrations obtained from the measuring station (out of 74 in the country) closest to the health clinic most frequently visited by the study participant were associated with a greater risk of dementia (Chang et al. 2014). In the Whitehall II longitudinal cohort study, PM exposure in London was modeled according to postcode for the years 2003–2009. Cross-sectional associations were observed between PM exposure and reasoning and memory, but not with verbal fluency. PM was also associated with a decline in cognition over time. However, the study provided no evidence for differential associations depending on particle source (Tonne et al. 2014). The results from two large cross-sectional studies in older adults in the United States suggested associations between fine particulate matter (PM_2.5_) and both cognitive function—primarily episodic memory (Ailshire and Crimmins 2014)—and error rates in cognitive assessments (Ailshire and Clarke 2015). These studies used a neighborhood-based measure of air pollution to represent the regional or urban background levels but did not account for variability related to local sources of pollutants.

There might also be short-term effects of air pollution on the brain that are related to the last few hours and days of exposure, and these might contribute to, or confound, associations with long-term exposure. As early as 1970, it was reported that mental efficiency decreased when adults tested in London were breathing air pumped from the street as opposed to clean air (Lewis et al. 1970). A recent study found that short-term exposure to ambient fine PM was associated with decreased resting cerebrovascular flow velocity and increased resting cerebrovascular resistance in community-dwelling elderly participants (Wellenius et al. 2013). In the present study, we investigated the relationship between exposure to air pollution and dementia incidence in Umeå, Sweden, using data from a prospective cohort study.

## Methods

*Participants.* Longitudinal data from the Betula study, described in detail elsewhere (Nilsson et al. 1997, 2004), were combined with individual traffic pollution data from the 50 m × 50 m grid where the participant’s home was located. The Betula study was initially motivated by the need to explore various aspects of health and cognitive aging, including early signs of and potential risk factors for cognitive decline and dementia in adulthood and late life. The Betula study has been carried out with a narrow age cohort design. At the first data collection in 1988–1990 (T1), an equal number of men and women, distributed among 10 age cohorts (all 35, 40, 45, … 80 years of age), were randomly sampled from the general population in the municipality of Umeå for a final sample of 1,000 participants. At the first follow-up, in 1993–1995 (T2), two new cohorts were included: sample 2 (S2; *n* = 995), with the same age and sex distribution as S1 at T1, and sample 3 (S3; *n* = 963), with the same age and sex distribution as S1 at the time of T2 (all 40, 45, 50, … 85 years). Data collections have, to date, been conducted six times with 5-year intervals between the follow-ups (T1, 1988–1990; T2, 1993–1995; T3, 1998–2000; T4, 2003–2005; T5, 2008–2010; T6, 2013–2014). S1 participants have been tested up to six times (T1–T6), and S3 participants have been tested up to five times (T2–T5). Data for S2 participants were collected at T2 and T3. At each test point (T1–T6), the investigation was split into two occasions, where a health survey constituted the first examination and a cognitive evaluation the second. Between the first and second testings, the study participants filled out a fairly extensive battery of self-assessment forms covering various socioeconomic, health, biological, aging, and personality traits. The health survey consisted of a health interview, health questionnaires, and blood (hemoglobin, blood glucose, sedimentation rate) and urine sampling for research purposes, as well as measurements of blood pressure, pulse, height, weight, waist–hip ratio (WHR), grip strength, sight, and hearing, and tests of odor identification. Research nurses or nursing assistants conducted the health survey according to a specified protocol. In the event of pathological findings, the study physician (R.A.) was consulted, and if deemed necessary, the participant was referred to his/her primary health care practitioner for follow-up.

The cognitive evaluations were conducted by investigators with basic training in psychology who were specially trained to carry out the Betula cognitive test battery. The battery of cognitive tests is extensive and covers a wide range of cognitive processes and memory systems to evaluate how normal memory changes with age. In addition, it is sufficiently powerful to identify and evaluate disease-related cognitive decline. The cognitive test battery includes tasks that assess episodic memory, semantic memory, working memory, the perceptual representation system, prospective memory, visual attention, processing speed, problem solving, and decision making. The MMSE and a questionnaire assessing subjective experiences of memory function and the subjective experience of memory loss are also included.

All participants in the Betula study gave informed consent, and the study was approved by the Regional Ethics Review Board at Umeå University with DNR: 2012-12-31M.

**Figure 1:**
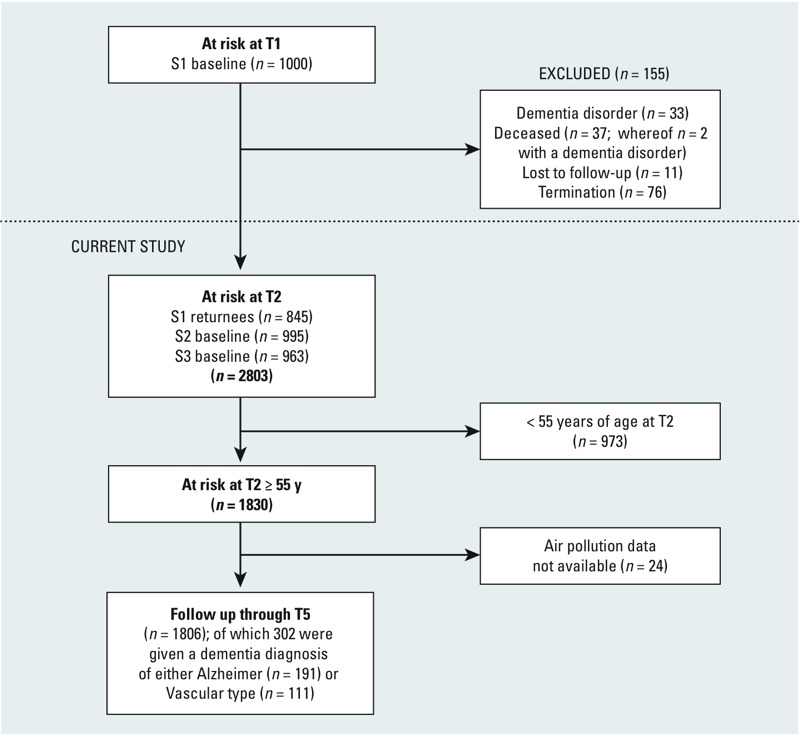
Flow chart from study inclusion to end of follow-up. ”Termination” refers to participants from S1 who did not want to, or could not, participate at T2, for example, because of illness.

In the present study, data on participants from samples S1, S2, and S3 gathered at T2 were considered baseline information (Figure 1). Participants in S1 who had died, had received a dementia diagnosis, were lost to follow-up, or had left the study for other reasons before T2 were excluded (*n* = 155). At the present study baseline (T2), the three samples consisted of 2,803 individuals without dementia; S1 (*n* = 845), S2 (*n* = 995), and S3 (*n* = 963). Participants > 55 years of age at T2 (*n* = 973) were thereafter excluded because of their low risk of developing dementia within the coming 15-year follow-up period. Participants with addresses that could not be geocoded (*n* = 24) were also excluded from analysis. Thus, the final sample consisted of 1,806 individuals (Figure 1).

*Dementia diagnoses.* In the Betula study, dementia status was assessed at baseline and reassessed every 5 years to identify new cases and to determine the year in which the DSM-IV (*Diagnostic and Statistical Manual of Mental Disorders, 4th Edition*) core criteria for dementia were met, that is, when cognitive symptoms became sufficiently severe to interfere with social functioning and with instrumental activities of daily living (American Psychiatric Association 2000). The diagnoses were based on observations obtained at the Betula study visits (health and cognitive evaluations) supplemented with medical record data covering the entire study period (1988–2010). Thus, the medical records from all hospital and primary care visits within the county were continuously evaluated. Available clinical results from magnetic resonance imaging, computerized tomography scans, and autopsy (not part of the Betula protocol), were also considered in the diagnostic decision. The Betula study population (*n* = 4,445) has been evaluated in this manner with regard to dementia after each test wave (T1, T2, T3, T4, T5). The diagnostic evaluations were coordinated by the same senior research geropsychiatrist (R.A.) throughout the study period. In 2011, an extensive quality assurance assessment of the dementia diagnoses in the Betula study was performed. In this assessment, a blinded reevaluation was made of the medical records of those with an established dementia diagnosis with regard to dementia status, subtype, and age at onset (Boraxbekk et al. 2015). In total, *n* = 444 individuals who had received a dementia diagnosis in previous evaluations were reevaluated by blinded investigators (i.e., the previous evaluation results were not given to the investigators performing the reevaluation), and dementia subtypes were distinguished between AD, vascular dementia (VaD), Parkinson dementia, Lewy body dementia, alcohol dementia, frontotemporal dementia, and dementia not otherwise specified. The reevaluated diagnoses were based on a minimum of 5 years additional information about the course of the disease.

As part of the Betula protocol, the following predetermined criteria, which were recorded at the health and cognitive examinations, were used as a guide for an extended evaluation as determined by the senior geropsychiatrist (R.A.): MMSE score ≤ 23, a decline in cognitive performance compared with a previous test occasion (from high to average/low or from average to low), a decline in daily functional activities or a subjective loss of memory function expressed by the participant in the semistructured interview, and any other behavioral or cognitive deviations, for example, confusion or disorientation noticed by the testing team. The high/low performance score was based on a composite cognitive measure, defined as a summed *z*-score of 1.8 above/below the mean of the age group. Participants in the intermediate range were categorized as “average” in terms of levels of cognitive performance. The composite cognitive measure was based on the principal component analysis presented by Nilsson et al. (1997). At test-waves T1 and T2, two geropsychiatrists participated in the evaluation process. At test-wave T3, four geropsychiatrists were involved in the diagnostic evaluation, thereby establishing a solid foundation for diagnostic consensus. In case of disagreement, the senior research geropsychiatrist (R.A.), who coordinated the diagnostic evaluations, was ultimately responsible for the final diagnosis.

Of the 1,806 participants included in the present study, 302 were diagnosed with either AD (*n* = 191) or VaD (*n* = 111) through T5. In this context, it should be noted that a dementia diagnosis given to a participant from the S1 or S3 cohorts was based both on clinical examinations and medical record data obtained between 1993 and 2011, whereas for the S2 participants, a dementia diagnosis given in T4 was based solely on information obtained from medical records.

*Study area and exposure measure.* The study area included Umeå municipality in northern Sweden, with an area of 2,397 km^2^ and a population of approximately 120,000 people in 2014. At baseline, the population was approximately 100,000. To describe the spatial contrasts in exposure to vehicle exhaust between the study participants, we geocoded the home addresses for all participants at baseline (1993–1995) using information from the Swedish population register. We developed a land-use regression (LUR) model for Umeå to estimate the annual average levels of nitrogen oxides (NO_x_), a commonly used marker of vehicle exhaust. The model was built around 36 measuring sites spread throughout the city of Umeå. At each site, between November 2009 and June 2010, 4 weeklong measurements were obtained with diffusive samplers (Ogawa samplers) to represent an annual average. For the sake of comparison with other air-pollution studies, we constructed our model using the same principles and geographical variables as in the large-scale European Study of Cohorts for Air Pollution Effects (ESCAPE; Beelen et al. 2013). The final LUR model explained 76% (adjusted *r*^2^ = 0.76) of the variation in measured NO_x_ values, and when validated using the “leave one out” cross-validation method, the adjusted *r*^2^ was 0.54. The LUR model was highly correlated (*r* = 0.86) for NO_x_ (and NO_2_) with the ESCAPE LUR model in Umeå, which was developed by the same team but used other, less extensive monitoring data.

We created a concentration grid (50 m × 50 m) covering the entire study area. Because some participants (1.6%) lived in rural areas outside the measurement area, the intercept was adjusted according to population density to avoid unrealistically high levels in rural locations. In a sensitivity analysis, the modeled NO_x_ values were rescaled using back-extrapolation to better correspond to the levels seen at the start of the follow-up period in this study (1993–1995).

The back-extrapolation was performed using information about the estimated total amount of traffic for each year and national vehicle fleet emission factors for Sweden based on the Artemis model (Sjödin et al. 2006). Using this information, we calculated scaling factors for each year, with 2009 as the baseline year.

**Figure 2:**
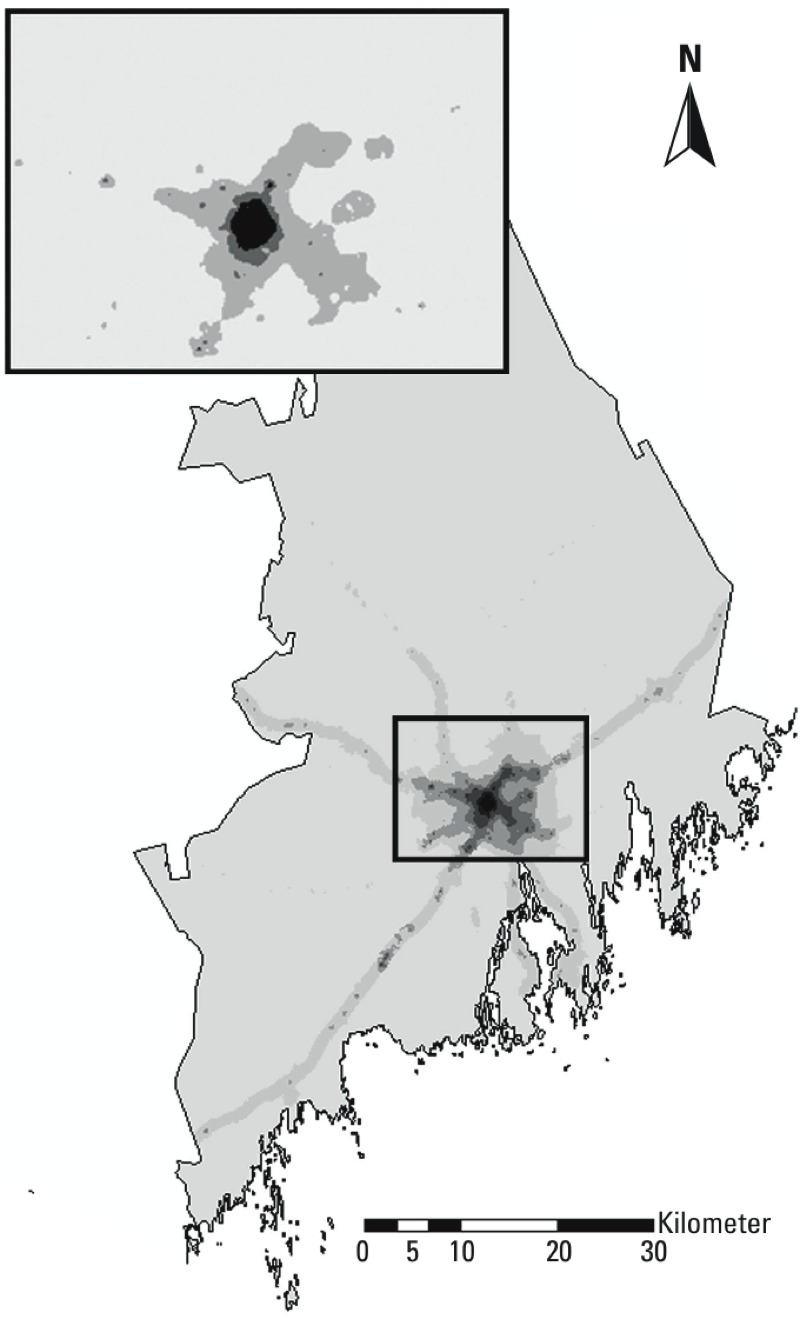
The study area of Umeå municipality illustrated with gradients of annual mean NOx concentration at the particpants’ home addresses at baseline. The lines of higher exposure radiating out from the central area (Umeå city center) correspond to major roads in the area. The smaller map shows the borders between the quartiles.

The estimated NO_x_ concentration at the home address at baseline was divided into quartiles (Figure 2). Participants in the lowest NO_x_ quartile had an annual mean exposure of < 9 μg/m^3^, the 2nd quartile had an annual mean exposure of 9 to < 17 μg/m^3^, the 3rd quartile had an annual mean exposure of 17 μg/m^3^ to < 26 μg/m^3^, and the 4th quartile had an annual mean exposure of ≥ 26 μg/m^3^, implying large contrasts among exposures within the study area. When rescaling the exposure with back-extrapolation, the quartile limits were 19, 35, and 54 μg/m^3^ for the 2nd, 3rd, and 4th quartiles, respectively.

*Potential confounding variables.* All potential confounders were defined according to the participant’s status at baseline (T2). In addition to age, there is moderate or strong evidence for associations between genetic, vascular, and psychosocial factors and the risk for dementia (Fratiglioni et al. 2008). In addition to age at baseline as a categorical variable, we considered several social and lifestyle variables as potential confounding variables (Table 1). Education, an indicator of socioeconomic status, was categorized as follows, depending on the highest education level achieved: elementary school, upper secondary school, and university level education.

**Table 1:** Distribution of dementia and population characteristics at baseline according to quartiles of NO_x_ exposure [*n* (%)].

Characteristic	NO_x_ Q1 4.8–9 μg/m^3^	NO_x_ Q2 > 9–17 μg/m^3^	NO_x_ Q3 > 17–26 μg/m^3^	NO_x_ Q4 > 26 μg/m^3^	All
All observations	433	476	459	438	1,806
Total dementia	55 (13)	65 (15)	90 (21)	92 (22)	302 (18)
Vascular dementia	23 (5)	22 (5)	31 (7)	35 (8)	111 (6)
Alzheimer’s disease	32 (8)	43 (10)	59 (14)	57 (14)	191 (11)
Sex
Male	205 (47)	205 (43)	189 (41)	174 (40)	773 (43)
Female	228 (53)	271 (57)	270 (59)	264 (60)	1,033 (57)
Age (years)
55	83 (19)	84 (18)	65 (14)	58 (13)	290 (16)
60	65 (15)	94 (20)	72 (16)	55 (13)	286 (16)
65	64 (15)	92 (20)	76 (17)	54 (13)	286 (16)
70	65 (15)	74 (16)	70 (15)	69 (16)	278 (15)
75	71 (16)	65 (14)	76 (17)	62 (14)	274 (15)
80	57 (13)	53 (11)	67 (15)	89 (20)	266 (15)
85	28 (6)	14 (3)	33 (7)	51 (12)	126 (7)
Education
Compulsory	360 (83)	354 (74)	344 (75)	345 (79)	1,403 (78)
High school	31 (7)	46 (10)	34 (7)	22 (5)	133 (7)
University	36 (8)	70 (15)	74 (16)	66 (15)	246 (14)
Missing	6 (1)	6 (1)	7 (1)	5 (1)	24 (1)
Physical activity
Never	91 (21)	90 (19)	105 (23)	126 (29)	412 (23)
Occasionally	57 (13)	61 (13)	57 (12)	44 (10)	219 (12)
A few times per month	38 (9)	46 (10)	39 (9)	53 (12)	176 (10)
Weekly	148 (34)	165 (35)	154 (34)	121 (28)	588 (33)
Daily	92 (21)	106 (22)	95 (21)	84 (19)	377 (21)
Missing	7 (2)	8 (2)	9 (2)	10 (2)	34 (2)
Smoking
Nonsmoker	236 (55)	278 (57)	249 (54)	238 (55)	1,001 (55)
Smoker	60 (14)	50 (11)	59 (13)	56 (13)	225 (12)
Former smoker	137 (32)	148 (31)	151 (33)	144 (33)	580 (32)
Alcohol
Yes	291 (67)	331 (70)	328 (71)	322 (74)	1,272 (70)
No, never	122 (28)	122 (26)	103 (22)	99 (23)	445 (25)
No, quit	19 (4)	21 (4)	28 (6)	17 (4)	85 (5)
Missing	1 (0.2)	2 (0.5)	0 (0)	1 (0.2)	4 (0.2)
BMI^*a*^
Overweight	293 (68)	318 (67)	288 (63)	281 (64)	1,180 (65)
Missing	8 (2)	22 (5)	15 (3)	15 (3)	60 (3)
WHR^*b*^
> Recommended	157 (36)	178 (37)	177 (39)	185 (42)	697 (39)
Missing	34 (8)	39 (8)	35 (8)	38 (9)	146 (8)
Hypertension	146 (34)	161 (34)	174 (38)	142 (32)	623 (34)
Missing	1 (0.2)	2 (0.4)	1 (0.2)	0 (0)	4 (0.2)
Diabetes	39 (9)	35 (7)	25 (5)	20 (5)	119 (7)
Missing	3 (0.7)	3 (0.7)	2 (0.5)	1 (0.2)	9 (0.5)
Stroke	18 (4)	28 (6)	33 (7)	38 (9)	117 (6)
Missing	2 (0.5)	3 (0.6)	2 (0.4)	1 (0.2)	8 (0.4)
ApoE4	114 (26)	115 (24)	130 (28)	135 (31)	494 (27)
Missing	11 (3)	21 (4)	16 (3)	13 (3)	61 (3)
Deceased	157 (36)	131 (28)	146 (32)	165 (38)	599 (33)
^***a***^Body mass index. BMI cut-offs between normal weight and overweight were 23.8 for women and 25.0 for men. ^***b***^Waist–hip ratio. Cut-offs were at 0.8 for women and 1.0 for men.					

Physical activity was assessed from interview data based on the participant’s answer to the following question: “During the last three months, did you do any sports, exercise, or walking?” The participant was allowed to choose from the following responses: “never,” “occasionally,” “a few times per month,” “weekly,” and “daily”; this question was used as a five-category variable in the analysis. Smoking status was categorized as present smoker, previous smoker, and nonsmoker at baseline. Alcohol consumption status was classified as present consumer, previous consumer, and nonconsumer at baseline. In previous studies, body mass index (BMI, kilograms per square meter) was associated with inflammatory effects associated with traffic pollution (Zeka et al. 2006) and with cognitive decline in women in the Betula cohort (Thilers et al. 2010). We therefore chose to include BMI as a potential confounding variable in the statistical analysis. Following National Institutes of Health standards from 1985 (Van Itallie 1985), the BMI cut-offs between normal weight and overweight were 23.8 for women and 25.0 for men. We also considered WHR as a potential confounding variable and used cut-offs of 0.8 for women and 1.0 for men (Nilsson and Nilsson 2009).

Diabetes, stroke, and hypertension are risk factors for dementia (Cheng et al. 2012; Sahathevan et al. 2012; Sharp et al. 2011) but might also be intermediate factors on the causal pathway between air pollution exposure and dementia. Therefore, we ran models with and without adjusting for a history of diabetes, stroke, and hypertension based on each participant’s status at baseline. In a study of overweight and cognition, it was considered necessary to include supplementary information on hypertension (Nilsson and Nilsson 2009). We followed the same criteria when defining hypertension in the present study: *a*) The participant reported the use of at least one of three blood pressure/heart medicines [Anatomic Therapeutic Chemical classification C01, C02, or C07 (Nilsson and Nilsson 2009)], or *b*) the systolic blood pressure was > 140 mmHg and/or the diastolic blood pressure was > 80 mmHg at the baseline (T2) health examination for inclusion in the Betula study. Additionally, because being an ε4 carrier of apolipoprotein E (ApoE4) adds substantially to at least the risk of developing AD or other forms of dementia (Anttila et al. 2002), the presence of at least one ε4 allele was taken into consideration in the analysis. Details on the genotyping methods have been published elsewhere (Nilsson et al. 2006).

*Statistical analyses.* Cox proportional hazards models were used with time as the underlying scale to calculate hazard ratios (HRs) and 95% confidence intervals (CIs) for dementia incidence in association with long-term exposure to air pollution. Censoring occurred with death, a diagnosis of dementia, loss to follow-up, or the end of follow-up, whichever came first. Censoring due to loss to follow-up occurred if a participant moved outside the Betula catchment area. Participants were censored if they were diagnosed with a dementia disorder of another subtype (for example, alcohol- or Parkinson-related dementia). No exact dates were available for the dementia diagnoses, only the year of onset. We therefore assumed onset to be 1 July for all participants with a dementia diagnosis. As a marker for long-term exposure to air pollution, we used the annual mean NO_x_ concentration (estimated for 2009–2010 using the LUR) at the residential address of the study participants at baseline. The NO_x_ concentration was used as a categorical variable in quartiles, with the first quartile as a reference category, and was tested as a continuous variable. We ran models adjusted for baseline age only (model 1); models adjusted for age and ApoE4, education, physical activity, smoking, sex, alcohol use, BMI, and WHR at baseline (model 2); and models that were further adjusted for the potential intermediate factors of hypertension, diabetes, and stroke (model 3), which were also defined at baseline (Table 1). In addition, we studied the two major diagnoses of AD and VaD separately. The end of follow-up was 30 June 2010.

In a subgroup analysis, we excluded participants who belonged to subsample S2—that is, who were younger than S1 and S3 and were only retested after 5 years—and in another analysis, we adjusted for sample (S1, S2, S3). In a sensitivity analysis, we excluded participants who were censored because they moved outside of the Betula catchment area before the end of follow-up. The validity of the inherent proportionality assumption was checked visually (by inspecting Kaplan–Meier plots) as well as with formal statistical testing. We included covariates in the model; these were entered as the interaction between the covariates and the natural logarithm of time. The time-dependent interaction terms were not statistically significant (*p* > 0.1), neither when tested individually nor when tested together, supporting the assumption of proportional hazards.

We used *a priori*–defined statistical models and backward elimination as a sensitivity analysis.

We calculated the population-attributable fraction, *AF_p_*, using the formula


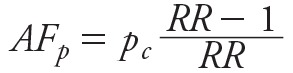
,

where *p_c_* is the exposure prevalence among cases, and RR is the relative risk associated with the exposure. We used the HR as a proxy of the RR in this setting, and we defined “exposed” as belonging to the 3rd or 4th quartile of NO_x_ exposure and “unexposed” as belonging to the 1st or 2nd quartile of NO_x_ exposure. We used the HR for dementia in association with exposure above versus below the median from the model adjusted for education, physical activity, smoking, sex, BMI, WHR, alcohol, age, and ApoE4 for the attributable fraction calculation. All analyses were performed using SAS for Windows, version 9.4 (SAS Institute Inc., Cary, NC).

## Results

During follow-up, 302 participants were diagnosed with either AD (*n* = 191) or VaD (*n* = 111). Twenty-nine participants were censored when they were diagnosed with a dementia disorder of another subtype, and 49 participants were censored when they moved from the catchment area and could no longer be followed in local medical records. The mean age at the end of follow-up was similar across exposure categories at 79, 79, 80, and 81 years in quartiles 1, 2, 3, and 4, respectively. Dementia risk from T2 (1993–1995) to 2010 (the cumulative dementia incidence) was similar across samples at 15% in S1, 17% in S2, and 18% in S3. The proportion of participants diagnosed with dementia increased with increasing NO_x_ concentration at the participant’s home (Table 1).

For the full cohort, participants in the group with the highest exposure were more likely than participants in the group with the lowest exposure to be diagnosed with dementia (HR = 1.43; 95% CI: 0.998, 2.05 for the highest vs. the lowest quartile; Table 2), whereas the HR associated with quartile 3 was 1.48 (95% CI: 1.03, 2.11; Table 2). When analyzed linearly, the exposure measure coefficients were not statistically significant (Table 2). Including hypertension, stroke, and diabetes in the statistical models had only a slight effect on the estimates (Table 2). The use of back-extrapolated exposure concentrations only marginally affected the estimates (data not shown). Adjusting for sample, or excluding participants who moved outside the Betula catchment area during follow-up, only marginally altered the HR estimates (data not shown). The assumptions regarding proportional hazards seemed to hold upon both visual inspection of the data and with formal statistical testing (data not shown). The HR associated with exposure (quartiles 3 and 4 vs. quartiles 1 and 2) was 1.37 (95% CI: 1.07, 1.76), and *p_c_* was 0.6, which resulted in an estimated population attributable fraction (*AF_p_*) of 16% (95% CI: 4, 26).

**Table 2:** Hazard ratios (HRs) and 95% confidence intervals (CIs) for dementia in association with baseline annual NO_x_ concentration from Cox proportional hazards models.

NO_x_^*a*^ (μg/m^3^)	*n* (person-years)	Cases	HR (95% CI)
Model 1^*b*^
4.8–9	433 (5,024)	55	1.0
> 9–17	476 (5,787)	65	1.10 (0.77, 1.58)
> 17–26	459 (5,182)	90	1.49 (1.07, 2.09)
> 26	438 (4,540)	92	1.57 (1.12, 2.19)
Per 10 μg/m^3^			1.04 (0.98, 1.11)
Model 2^*c*^
4.8–9	381 (4,553)	50	1.0
> 9–17	420 (5,286)	59	1.11 (0.76, 1.63)
> 17–26	408 (4,700)	82	1.48 (1.03, 2.11)
> 26	383 (4,037)	84	1.43 (0.998, 2.05)
Per 10 μg/m^3^			1.05 (0.98, 1.12)
Model 3^*d*^
4.8–9	378 (4,538)	49	1.0
> 9–17	418 (5,264)	59	1.13 (0.77, 1.66)
> 17–26	406 (4,677)	81	1.49 (1.04, 2.14)
> 26	383 (4,037)	84	1.60 (1.02, 2.10)
Per 10 μg/m^3^			1.05 (0.98, 1.12)
^***a***^The annual non–back-extrapolated NO_x_ baseline concentration at the residence of each participant was used as a proxy for exposure to air pollution. ^***b***^Model 1: age-adjusted. ^***c***^Model 2: adjusted for baseline age, education, physical activity, smoking, sex, BMI (body mass index), WHR (waist–hip ratio), alcohol, and ApoE4. ^***d***^Model 3: model 2 plus baseline medical history of diabetes, hypertension, and stroke.			

When the sample that was retested only after 5 years (S2) was excluded, the number of events decreased from 275 to 181, the number of study subjects decreased from 1,592 to 1,065, and the number of person-years decreased from 18,576 to 12,295. In that sample, participants in the group with the highest exposure were more likely than participants in the group with the lowest exposure to be diagnosed with dementia (HR = 1.71; 95% CI: 1.08, 2.73 for the highest vs. the lowest quartile; Table 3).

**Table 3:** Hazard ratios (HRs) and 95% confidence intervals (CIs) for dementia in association with baseline annual NO_x_ concentration from Cox proportional hazards models (sample 2 excluded).

NO_x_^*a*^ (μg/m^3^)	*n *(person-years)	Cases	HR (95% CI)
Model 1^*b*^
4.8–9	262 (3,065)	32	1.0
> 9–17	317 (3,793)	45	1.24 (0.79, 1.96)
> 17–26	320 (3,625)	57	1.49 (0.97, 2.30)
> 26	315 (3,172)	67	1.72 (1.13, 2.62)
Per 10 μg/m^3^			1.05 (0.98, 1.13)
Model 2^*c*^
4.8–9	227 (2,757)	27	1.0
> 9–17	275 (3,423)	40	1.39 (0.85, 2.28)
> 17–26	285 (3,294)	52	1.58 (0.98, 2.53)
> 26	278 (2,821)	62	1.71 (1.08, 2.73)
Per 10 μg/m^3^			1.08 (1.00, 1.16)
^***a***^The annual non–back-extrapolated NO_x_ baseline concentration at the residence of each participant was used as a proxy for exposure to air pollution. ^***b***^Model 1: age-adjusted. ^***c***^Model 2: adjusted for education, physical activity, smoking, sex, BMI (body mass index), WHR (waist–hip ratio), alcohol, age, and ApoE4.			

The analysis stratified by diagnosis yielded estimates of similar magnitude for AD and VaD, with an HR for AD of 1.38 (95% CI: 0.87, 2.19; Table 4) and an HR for VaD of 1.47 (95% CI: 0.83, 2.61; Table 4) for the highest versus the lowest quartile.

**Table 4:** Hazard ratios (HRs) and 95% confidence intervals (CIs) for Alzheimer’s disease and vascular dementia in association with baseline annual NO_x_ concentration from Cox proportional hazards models.

NO_x_^*a*^ (μg/m^3^)	*n* (person-years)	Cases	HR (95% CI)
Alzheimer’s disease
Model 2^*b*^
4.8–9	381 (4,553)	30	1.0
> 9–17	420 (5,286)	39	1.15 (0.72, 1.86)
> 17–26	408 (4,700)	52	1.51 (0.96, 2.37)
> 26	383 (4,037)	52	1.38 (0.87, 2.19)
Per 10 μg/m^3^			1.05 (0.97, 1.15)
Vascular dementia
Model 2^*b*^
4.8–9	381 (4,553)	20	1.0
> 9–17	420 (5,286)	20	1.15 (0.62, 2.11)
> 17–26	408 (4,700)	30	1.46 (0.83, 2.61)
> 26	383 (4,037)	32	1.47 (0.83, 2.61)
Per 10 μg/m^3^			1.02 (0.92, 1.14)
^***a***^The annual non–back-extrapolated NO_x_ baseline concentration at the residence of each participant was used as a proxy for exposure to air pollution. ^***b***^Model 2: adjusted for education, physical activity, smoking, sex, BMI (body mass index), WHR (waist–hip ratio), alcohol, age, and ApoE4.			

## Discussion

Our results suggest an association between air pollution exposure and dementia incidence, with an HR of 1.43 (95% CI: 0.998, 2.05) for the highest versus the lowest quartile of exposure; the HR was higher in the subanalysis that excluded the younger sample that was retested only after the first 5 years of the follow-up period (HR = 1.71; 95% CI: 1.08, 2.73). Our findings are supported by a growing number of epidemiological studies showing an association between air pollution exposure and cognitive impairment in the elderly (Ailshire and Clarke 2015; Ailshire and Crimmins 2014; Chang et al. 2014; Chen and Schwartz 2009; Power et al. 2011; Ranft et al. 2009; Tonne et al. 2014; Wellenius et al. 2012; Weuve et al. 2012a) and by toxicological studies suggesting that air pollution has neuropathological effects (Block and Calderón-Garcidueñas 2009). It should be noted that the sizes of the estimates associated with quartiles 3 and 4 are similar and that we observed no statistically significant linear association with the exposure measure. Interestingly, Ailshire and Crimmins (2014) observed a similar tendency when they studied cognitive function and PM_2.5_, with a higher risk in quartile 3 than in quartile 4 (although they did not observe a statistically significant difference). We were not able to control for the floor location of the residence (i.e., in multistory buildings), infiltration of outdoor air pollutants, exposure to wood smoke, or time spent away from the residence, any or all of which may have resulted in exposure misclassification.

Previous studies on air pollution and aging have used cross-sectional data or have been based only on cognitive testing, and studies on cognitive outcomes are especially susceptible to bias from selective attrition (Weuve et al. 2012b).

The present study has a major strength in the longitudinal high-quality data from the Betula study and the dementia diagnoses, specified by subtype and age at onset. There are often difficulties in elucidating etiology/pathophysiology in dementia disorders, particularly if a patient develops dementia at an advanced age; these difficulties are mainly due to an increasing burden of vascular complications. In the Betula study, the primary aim of the diagnostic process was to determine a clinical diagnosis of dementia and to be confident that we had identified a progressive course of the disorder. We adopted a conservative taxonomic approach, that is, establishment of a diagnosis of AD or VaD or other, less common dementia disorders such as frontotemporal lobe dementia, Lewy body dementia, or Parkinson dementia. The concept of AD/VaD mixed-type dementia was not of primary interest, mainly owing to the high prevalence of risk factors for cardiovascular disease (CVD) that are present in persons of advanced age.

Clinically, significant CVD risk factors were recorded during the entire follow-up period, and presence of these factors favored a diagnosis of VaD; however, this diagnosis was made only when these risk factors were sufficiently severe and were combined with neurological signs and symptoms of VaD. It is of importance to stress that the mere presence of CVD risk factors did not automatically lead to a diagnosis of VaD because these risk factors are also of etiological relevance in AD (e.g., hypertension, diabetes mellitus, hypercholesterolemia). The vascular types of lesions most likely also contribute to AD pathology and/or exert additive adverse effects on cognition; however, this hypothesis continues to be debated (Attems and Jellinger 2014). In autopsy studies comparing VaD and AD/VaD mixed-type dementia, a similar distribution and frequency of multiple infarcts, subcortical lacunae, and strategic infarcts was reported (Jellinger 2013). Not unexpectedly, clinicopathological studies using the current criteria for VaD show only moderate sensitivity. Thus, there is also a dilemma involved with using the strictly taxonomic approach (AD, VaD, AD/VaD mixed-type dementia, and other dementias). Accordingly, it was recently proposed that the complexity encountered in determining the role of CVD in VaD, AD, and AD/VaD mixed-type dementia should be replaced by a more integrative approach that takes multiple pathophysiological mechanisms into account (Kling et al. 2013). Furthermore, there is a lack of clinical and histopathological consensus regarding the impact of cerebrovascular disorders necessary for a “pure” VaD or AD/VaD mixed-type diagnosis, which gives rise to multilevel diagnostic difficulties.

A strength of the present study is the fine-scale LUR model, which had a high level of precision (adjusted *r*^2^ = 0.76), was constructed according to the ESCAPE protocol, and offered good external validity (Beelen et al. 2013). Most previous studies on cognitive outcomes and air pollution used a neighborhood-based measure of air pollution. In such studies, it is difficult to investigate the association with traffic emissions near the home. The exposure measure in the present study, which modeled exposure at the home address at baseline, is prone to exposure misclassification because in reality, exposure to air pollution is not restricted to when a person is at his/her home. A limitation of the present study is that we used information on the home address at inclusion, which, however unlikely, would create a false association.

Residual confounding, for example, from traffic noise, is a possible explanation for our findings. We considered several potential confounding factors, but only adjustment for age had any substantial influence on the association between traffic pollution and dementia incidence. Factors related to socioeconomic status, such as education and smoking, did not influence the observed association. The potential intermediate factors of stroke, hypertension, and diabetes did not generally influence the estimates (Table 2). Interestingly, the estimates became higher when sample S2 (retested only once, after 5 years) was excluded. Dementia incidence was similar across samples. Therefore, diagnostic bias due to fewer cognitive testing occasions does not explain the seemingly higher HRs observed when S2 was removed. Sample S2 had the lowest proportion of participants in the quartile with the highest exposure (20%), perhaps because of the younger age distribution, which might be a partial explanation.

Umeå is a city with very low regional background levels of air pollution, but there are strong gradients within the city, and yearly violations of the NO_2_ limits are reported to the Swedish Environmental Protection Agency and the European Environmental Agency. The recorded levels of NO_2_ at monitoring stations fell over the course of the study period (Naef and Xhillari 2001), but the spatial patterns were assumed to be constant. Back-extrapolation of exposure levels only marginally altered the estimates, which is not surprising because baseline exposure was limited to three different years (1993–1995). The major source of our exposure measure, NO_x_, was vehicle exhaust from local traffic. However, road dust shares the same patterns as those of NO_x_ and exhaust particles in Sweden (Johansson et al. 2007).

## Conclusions

We observed associations between dementia incidence and local traffic pollution that remained after adjusting for known risk factors. The magnitude of the association was similar for both AD and VaD. However, residual confounding from other environmental factors such as traffic noise and other potential sources of bias cannot be ruled out. The results of the present study are thus a strong indication of the need for further research using prospective cohorts.
